# Supplementary material for: Content-rich biological network constructed by mining PubMed abstracts
Source: BMC Bioinformatics. 2004 Oct 8;5:147. doi: 10.1186/1471-2105-5-147 (PMC528731; doi:10.1186/1471-2105-5-147)
Supplement: Additional File 2 — The original results of the above study (non-essential files are deleted to keep the file size under the limit set by BMC bioinformatics). [file 1471-2105-5-147-S2.bz2 › chilibotAdditionalFile2/dip20/412ID10712517E10078/html/BMPR1B_BMPR1A.html]

 


 **BMPR1B** and **BMPR1A** 
  
Found 25 abstracts in PubMed, retrieved 20.  
 

 What does Google say? 
 PDF only 
| .edu only 

---

**Interactive relationship** (e.g. stimulation, inhibition, etc)

**Neutral relationship**- BMP 4 and GDF 5 are known to bind to activin receptor like kinase 3  [ **BMPR1A** ]  ALK 3  [ **BMPR1A** ]  and or ALK 6  [ **BMPR1B** ]  also termed BMP type IA and type IB receptors, respectively.  Ref: 11282024 J Cell Sci, 2001
- However, endoglin binds BMP 2 by interacting with the ligand binding type I receptors, ALK3  [ **BMPR1A** ]  and ALK6  [ **BMPR1B** ] .  Ref: 9872992 J Biol Chem, 1999
- Constitutively active BMP type I receptors, ALK 3  [ **BMPR1A** ]  and ALK 6  [ **BMPR1B** ] , as well as ALK 2, were phosphorylated Smad8 and induced Smad8 interaction with Smad4.  Ref: 10814522 Biochem Biophys Res Commun, 2000
- The ALK 3  [ **BMPR1A** ]  mRNA began to appear in this stage, as did ALK 6  [ **BMPR1B** ]  and BMP RII.  Ref: 11147870 J Neurosurg, 2001

**Non-interactive relationship** (e.g. studied together, co-existance, homology, etc.)

- A mechanistic explanation for increased pSmad1 5 staining during inflammation was provided by the upregulated expression of all the BMP type I receptors, i.e., activin receptor like kinase ALK 2, ALK3  [ **BMPR1A** ] , and ALK6  [ **BMPR1B** ] , in the inflamed bronchial epithelial cells.  Ref: 12151307 Am J Respir Cell Mol Biol, 2002
- Using somatic cell hybrid and FISH analyses, the  **BMPR1A** ,  **BMPR1B** , and BMPR2 genes were assigned to 10q2 q22 24, and 2q33 34, respectively.  Ref: 10051328 Mamm Genome, 1999
- BMP type II receptor BMPR II is a type II serine threonine kinase receptor, which transduces signals for BMPs through heteromeric complexes with type I receptors, including activin receptor like kinase 2 ALK2, ALK3  [ **BMPR1A** ]  BMPR IA, and ALK6  [ **BMPR1B** ]  BMPR IB.  Ref: 10772805 Dev Biol, 2000
- Thus, ALK 2 and ALK 3  [ **BMPR1A** ]  or ALK 6  [ **BMPR1B** ]  might synergistically induce osteoblast differentiation of C2C12 cells, possibly through efficient activation of downstream signaling pathways.  Ref: 11282024 J Cell Sci, 2001
- ALK 2, ALK 3  [ **BMPR1A** ]  and ALK 6  [ **BMPR1B** ]  mRNA expression increased with the progression of PDL cell differentiation, suggesting that these receptors may play important roles in Dex induced PDL cell differentiation and mineralized nodule formation.  Ref: 10633885 J Periodontal Res, 1999
- ... bone morphogenetic protein BMP type I receptor genes are transcribed either ubiquitously Alk3  [ **BMPR1A** ]  or exclusively in the WD and ureter epithelium Alk6  [ **BMPR1B** ] .  Ref: 10749566 J Clin Invest, 2000
- Mouse smad8 phosphorylation downstream of BMP receptors ALK 2, ALK 3  [ **BMPR1A** ] , and ALK 6  [ **BMPR1B** ]  induces its association with Smad4 and transcriptional activity.  Ref: 10814522 Biochem Biophys Res Commun, 2000
- Messenger RNAs for BMP, 7, the BMP type I receptors Act R 1 alk 2, activin like kinase receptor, BMPR IA alk 3  [ **BMPR1A** ] , IB alk 6  [ **BMPR1B** ] , and the type II receptor BMPR II were detected.  Ref: 11829442 Biomaterials, 2002
- Compared with the effects induced by only one of the type I receptors, the combination of constitutively active forms of ALK 2 and ALK 3  [ **BMPR1A** ]  or ALK 6  [ **BMPR1B** ]  more strongly induced alkaline phosphatase activity in C2C12 cells.  Ref: 11282024 J Cell Sci, 2001
- We then investigated whether the sensitivity of the early long bone rudiments to OP 1 correlated with high expression of the OP 1 binding type I serine threonine kinase receptors activin receptor like kinase ALK 2 ActR I, ALK 3  [ **BMPR1A** ]  BMPR IA or ALK 6  [ **BMPR1B** ]  BMPR IB at this early stage.  Ref: 10705576 Growth Factors, 2000
- We assessed the distribution and relative immunohistochemical staining intensity of the bone morphogenetic protein 7, osteogenic protein 1 OP 1, in its pro and mature forms, and four of its receptors, type I ALK 2, ALK 3  [ **BMPR1A** ] , and ALK 6  [ **BMPR1B** ]  and type II in normal adolescent New Zealand White rabbit articular cartilage.  Ref: 12364567 J Histochem Cytochem, 2002
- We analyzed poly RNA from rat incisor pulp and bovine adult pulp by reverse transcriptase polymerase chain reaction, using a degenerate primers corresponding to the most conserved amino acid sequences in the intracellular serine threonine kinase of type I or type II like kinase 1, ALK 2, ALK 3  [ **BMPR1A** ] , ALK 4, ALK 5, ALK 6  [ **BMPR1B** ] , and BMPR II was found to be in dental pulp.  Ref: 9294489 J Dent Res, 1997
- Activin receptor like kinase ALK 1, ALK 2, ALK 3  [ **BMPR1A** ] , and ALK 6  [ **BMPR1B** ]  were able to reproduce the hypertrophic maturation of chondrocytes induced by BMP 2.  Ref: 12082094 J Biol Chem, 2002
- No homozygous deletions were detected in the ALK 1, ALK 2, ALK 3  [ **BMPR1A** ] , or ALK 6  [ **BMPR1B** ]  genes in a panel of 86 pancreatic cancer xenografts and 11 pancreatic cancer and 22 breast cancer cell lines.  Ref: 9850059 Cancer Res, 1998
- Populations of rare CD34 CD38 Lin stem cells were isolated from human hematopoietic tissue and were found to express the BMP type I receptors activin like kinase ALK 3  [ **BMPR1A** ]  and ALK 6  [ **BMPR1B** ] , and their downstream transducers SMAD, and 5.  Ref: 10190905 J Exp Med, 1999
- The induction of Tlx2 luc was dependent upon the kinase activity of MISRII and was blocked by a dominant negative truncated ALK2 tALK2 receptor but not by truncated forms of the other BMP type I receptors ALK1, ALK3  [ **BMPR1A** ] , or ALK6  [ **BMPR1B** ] .  Ref: 11376113 Mol Endocrinol, 2001
- Alk3  [ **BMPR1A** ]  tended to decrease, but Alk6  [ **BMPR1B** ]  remained unchanged.  Ref: 11675415 J Am Soc Nephrol, 2001
- The sections were rehydrated, submitted to microwave antigen retrieval and treated with polyclonal antibodies against  **BMPR1A** ,  **BMPR1B**  and BMPR2.  Ref: 11882013 Reproduction, 2002
